# The elements of resilience in the food system and means to enhance the stability of the food supply

**DOI:** 10.1007/s10669-022-09889-5

**Published:** 2023-01-02

**Authors:** Rimhanen Karoliina, Aakkula Jyrki, Aro Kalle, Rikkonen Pasi

**Affiliations:** 1 Bioeconomy and Environment, Natural Resources Institute Finland (Luke), Latokartanonkaari 9, 00790 Helsinki, Finland; 2 Bioeconomy and Environment, Natural Resources Institute Finland (Luke), Lönnrotinkatu 7, 50100 Mikkeli, Finland

**Keywords:** Social-ecological system, Shocks, Changes, Food security, Resilience, Food system, Policy coherency, Finland

## Abstract

Food systems are increasingly exposed to disruptions and shocks, and they are projected to increase in the future. Most recently, the war in Ukraine and Covid-19 pandemic has increased concerns about the ability to secure the availability of food at stable prices. This article presents a food system resilience framework to promote a national foresight system to better prepare for shocks and disruptions. Our study identified four key elements of resilience: system thinking through science and communication; redundancy of activities and networks; diversity of production and partners; and buffering strategies. Three national means to enhance resilience in the Finnish food system included domestic protein crop production, renewable energy production, and job creation measures. Primary production was perceived as the cornerstone for food system resilience, and the shocks and disruptions that it confronts therefore call for a sufficient and diverse domestic production volume, supported by the available domestic renewable energy. A dialogue between different actors in the food system was highlighted to format a situational picture and enable a rapid response. Our study suggests that to a certain point, concentration and interdependence in the food system increase dialogue and cooperation. For critical resources, sufficient reserve stocks buffer disruptions over a short period in the event of unexpected production or market disruptions. Introducing and strengthening the identified resilience elements and means to the food system call for the preparation of a more holistic and coherent food system policy that acknowledges and emphasises resilience alongside efficiency.

**Supplementary Information:**

The online version contains supplementary material available at 10.1007/s10669-022-09889-5.

## Introduction

Food systems are increasingly exposed to disruptions and shocks, and these are projected to increase in the future (van der Vegt et al. 2015; Maye et al. 2018). The recent Covid-19 pandemic has stirred actors to promote food system resilience (Devereux et al. 2020; Laborde et al. 2020) to ensure the availability of and access to nutritious and safe food, despite unexpected disruptions in the operating environment. The complexity of interactions between change factors, processes, actors, and different sectors increases the vulnerability of the food system to sudden disruptions (Ericksen 2008). Identifying the key elements and means of resilience would advance the food system’s anticipation of unexpected shocks and changes, improving the national foresight system to better support short- and long-term decision-making in future disruptions.

Resilience is related to the ability of a system to maintain its structure and functions, and when necessary, to adapt and reorganise in the face of disruptions (Holling 1973; Holling and Gunderson 2002; Folke 2006; Folke et al. 2010; Bullock et al. 2017). Arising from ecology (Holling 1973), the theoretical constructs of resilience aid the understanding of the dynamics and functioning of many types of social-ecological systems, including food systems (Tendall et al. 2015; Bullock et al. 2017; Stone and Rahimifard 2018).

Food systems include the people and activities involved in producing, transporting, supplying, and consuming food (Food Systems Dashboard 2020). In this study, the focus is on the food supply chain, including the steps of agricultural production, storage and distribution, processing, packaging, and retail. The activities in the food supply chain are strongly connected with each other, and also with social wellbeing and natural capital, including geology, soils, air, water, and all living things, as well as ecosystem services and through feedback mechanisms, to drivers controlling food system operations (Ericksen 2008). Tendall et al. (2015) defines food system resilience as the “capacity over time of a food system and its units at multiple levels, to provide sufficient, appropriate and accessible food to all, in the face of various and even unforeseen disturbances”.

Multidimensional changes in the operating environment demand complex food systems not only to prevent shocks, but to develop adaptive capacity to adjust to continuous changes (Tendall et al. 2015). In such systems, resilience is manifested as cyclical and continuous adaptation and learning caused by changes and disruptions (Holling and Gunderson 2002). Meuwissen et al. (2019) identified three capacities of resilience: robustness, which is related to a system’s capacity to resist and withstand change; adaptability, which refers to a system’s ability to adjust its operations in response to change; and transformability, which is the capacity to change internal structures and operations in response to change (Meuwissen et al. 2019; Holling and Gunderson 2002). In addition, the recovery from a disruption is important for overcoming challenges (Hollnagel et al. 2011; Linkov and Trump 2019).

Agriculture in Finland is characterised by its northern climate and self-sufficiency in most major agricultural products (Niemi and Väre 2019). The economic role of agriculture is declining in terms of GNP and employment in primary production, but with the food industry and forestry, it constitutes a significant part of the Finnish economy: 12% of employed people and 17% of output (Torvelainen et al. 2020). Agriculture employed 64,300 people, and with the food sector, 104,100 people, in 2019 (Torvelainen et al. 2020). In primary production, the number of farms more than halved between 1995 and 2021, amounting to 45,630 agricultural and horticultural enterprises in 2020, and in the same period, the average farm size increased from 22 to 50 ha (OSF 2021). Regional variation in the production structure and between agricultural branches (livestock, crop, and horticulture production) is considerable, and production lines are regionally concentrated. Finnish agriculture is almost exclusively based on family farms—some 86% of all farms—while farming syndicates and farms owned by heirs and limited liability farms represent about 9% and 3% respectively. Farm size is largest in Southern Finland and smallest in Eastern Finland. Almost half the arable land is in Southern Finland. The amount of arable land has been quite stable—a total of almost 2.3 million hectares. Primary production has struggled with poor profitability for the last 10 years (Economy Doctor 2021).

The two main sectors in the Finnish food industry are the dairy and meat processing industries. Together, they accounted for 43% of the food industry’s turnover in 2016 (Niemi and Väre 2019). In the Finnish retail sector, the consolidation trend has continued for an extended period, resulting in the two largest chains having a market share of around 80% in the 2010s (Niemi and Väre 2019). Foodstuffs are consumed mainly domestically. Traditionally, more than half of Finnish food exports have gone to neighbouring countries, but following the Russian import embargo, the share decreased dramatically. In 2017, the neighbouring countries’ combined share of food exports was just over 40% (Sweden 19.5%, Estonia 10%, and Norway 2.8%) (Niemi and Väre 2019). As an EU member state, Finland belongs to the EU’s common agricultural policy (CAP), which aims to (1) support farmers and improve agricultural productivity, ensuring a stable supply of affordable food, (2) safeguard a reasonable living for farmers, (3) help tackle the impacts of climate change and the sustainable management of natural resources, (4) maintain rural areas and landscapes across the EU, and (5) keep the rural economy alive by promoting jobs in farming, agri-food industries, and associated sectors (EC 2021). These general long-term goals also support the maintenance of system resilience, but sudden shocks such as Covid-19 can directly affect goals one and five, and the other goals indirectly.

So far, Finland lacks comprehensive food system policy. Instead, the development of parallel policies guiding and affecting food system development has been the case (e.g. CAP, Rural policy, Climate and energy policy, trade policy) see Rikkonen 2017, Himanen et al. 2016. The Ministry of Agriculture and forestry (MAF 2021) has defined the food policy in a comprehensive way including the overall food system from primary production to consumption, citizen health and environmental and climate issues, but the implementation of food policy as such exists only through established policies such as CAP, rural policy, climate and energy policy etc. However, systemic approach has been highlighted in food strategy work (MAF 2021), but concrete measures to form a shared food policy has started just in recent years (see Kaljonen et al 2022).

In Finland, the maintenance of society´s critical operations are not left solely to the markets. The National Emergency Supply Organisation (NESO) secures society´s critical operations, including the food supply, together with public, private and third sector. The state and municipal authorities have a statutory obligation to prepare for exceptional situations, while companies are as a rule involved voluntarily. The NESO for example agrees on grain storage with food industry to secure sufficient buffer (NESO 2022).

Under normal circumstances, Finnish agriculture can meet the needs of domestic consumption well. Finland is self-sufficient in the production of cereals, meat, and milk but the production of oil and protein crops is in deficit (Jansik et al. 2021). However, self-sufficiency in agriculture is not a sufficient measure for food security. Notably quantities of production inputs are imported to Finland which increases the dependence on the availability of imported inputs (Jansik et al. 2021).

Resilience studies have increased extensively in recent years (Pettit et al. 2010; Tendall et al. 2015; Kamalahmadi and Parast 2016; Stone and Rahimifard 2018). These studies have identified elements which control resilience and can be used in management to reduce vulnerabilities. Diversification across the food supply chain and at different levels has been highlighted as one of the key elements of resilience (Hertel et al. 2021). In a literature review, Stone and Rahimifard (2018) identified 40 resilience elements, categorising them as “core” and “supporting” elements. In the context of supply chain resilience, collaboration, flexibility, agility, visibility, and adaptability were the most frequently cited elements. Tendall et al. (2015) emphasised the importance of including social, economic, and biophysical processes operating at different scales of the examined food system level. However, there is a gap in the existing literature on what concrete means help food system actors manage resilience. The diversity of operating environments and actors make the key elements and strategies very case specific. In this study, we therefore aimed to increase the understanding of the key elements and means to enhance resilience in the Finnish food system. We defined the key elements as system characteristics which are suggested to enhance the resilience, and the means as concrete measures actors can manage.

We formulated the following research questions in this study:

1. What are the main shocks and changes the Finnish food system in the 2020s faces, and how do they affect food security?
2. What are the key elements and means contributing to resilience, and how do they do so?

## Materials and methods

### Expert interviews

The material in this study consists of nine semi-structured in-depth interviews with Finnish food systems and food security experts. Experts with different backgrounds regarding the food system’s function (primary production, processing, trade, and administration), scale (local, global), and expertise (scientific and practical knowledge) were selected for interview. The professional status of the experts represented research and development (2), National emergency supply (3), interest organization (2), government officials (1), industry and trade (1). The interviews were conducted in November–December 2021 using Microsoft Teams remote access, following the physical distancing recommendation during Covid-19. The interviews were recorded and transcribed. Each interview lasted about one and a half hours. The interviews’ themes included open questions concerning the main disruptions and changes faced by the Finnish food system in the 2020s, the impacts of disruptions on the stability of the food supply, and enablers of and barriers to preparing for change (Supplementary information 1). The interview guide included a part in which interviewees were asked to judge given changes according to their importance for food security (Supplementary information 1). The impacts of disruptions and preparedness were approached through examples of disruptions which interviewees could select from a list without restrictions.

### Analysis

**Fig. 1:**
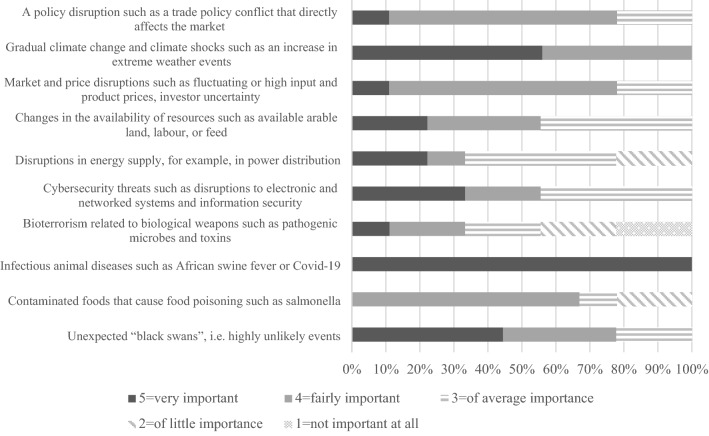
Expert evaluation of the importance of different disruptions for Finnish food systems (scale from 1 to 5)

The data were analysed qualitatively using relational content analysis with inductive approach (Krippendorff 2004; Elo et al. 2014). The unit of analysis was a meaningful thought separated from the text of individual experts. The transcribed interview material was first coded and condensed to distinguish general themes (Tables 1 and 2) using NVivo Qualitative Data Analysis Software 1.3. Coding and condensing were fully based on the data. The analysis focused on interpreting the meaning of the content, not content frequencies. The themes were further interpreted, structured and compacted to more general system elements by examining the data through resilience theory (Fig. 1 and Table 3). From the data, we identified three nationally relevant concrete means to enhance the resilience through interpreting emphases of the experts. We identified such means as promoting the removal of currently critical bottlenecks and high impact for the food system resilience. We defined the resilience of the Finnish food system as the ability to secure the food supply despite shocks and disruptions. In the analysis, we sought answers to questions related to,em elements and the concrete means actors could implement to enhance food system resilience.

**Table 1:** Critical disruptions and impacts on food supply security

Disruption	Impact on food supply security
Extreme weather events	A decline in the availability of domestic raw materials, influencing food processing and exports Economic losses in primary production Rising consumer prices
Market disruptions	The shutdown of the food industry and exports A decline in sales for companies producing inputs Economic losses at all food system levels Declining availability of imported protein feed Endangered animal welfare Cold chain breakage of perishable products Loss of reputation and trust of business partners Rising consumer prices
Infectious animal diseases	Economic losses in primary production Collapse of meat production Endangered animal welfare due to prevented access to processing The shutdown or reorganisation of the food industry Market disruptions Lack of domestic raw materials in industry Stagnation of exports Declining access to animal products for consumers Rising consumer prices Collapse in consumer confidence Export ban Financial losses at all food system levels
Disruptions in the availability of foreign labour	A labour shortage on farms A lack of domestic raw materials in industry Rising prices for industry and consumers
Disruptions in the energy supply	Shutdown of information systems Inoperative processes at all levels of food system and in distribution Disruptions in payment systems Financial losses at all food system levels
Cyber incident	Shutdown of processes and production plants Endangered animal welfare due to prevented access to processing Reduced selection of food for consumers Financial losses at all food system levels
Bioterrorism Contaminated foods	Reduced food safety Collapse in consumer confidence Reduced selection of food for consumers Financial losses at all food system levels
Black swans	Unexpected widespread impacts and feedbacks

**Table 2:** Identified means to enhance resilience at different levels of the food system

Level of food system	Mean
Primary production	Employee support in year-round production such as dairy Diversity in production, crops, varieties, and crop rotations Investment in field growing conditions, drainage, irrigation systems, and nutrient recycling Increasing self-sufficient bioenergy production Acquisition of reserve electricity Farm storages Farm-scale biogas plants Hygiene Contingency plans, self-monitoring, biosecurity Investment in information security Education, communication
Processing	Food and input imports Acquisition of reserve electricity Contract production Binding prices in production season and yield Restrictions on imports of meat products from high-risk countries Alternative market channels Division of workers, encapsulation of work shifts Diversion of raw material flows to other units and processes Self-monitoring, communication Storage Hygiene Contingency plans, self-monitoring, biosecurity Investment in information security Education, communication
Retail trade	Contracts Compensatory products Decentralised retail network Acquisition of reserve electricity and information security, encapsulation of work shifts, hygiene Investment in information security Education, communication
Society	Production surplus relative to consumption Increasing domestic production of critical inputs that are currently imported Wide geographical coverage of domestic production and processing plants Plant breeding Agricultural research Location of critical operations in areas of best secured electricity networks Diversity of production sectors Reserve stocks for critical inputs and food Improving labour mobility Breaking down incentive traps for potential labour Quarantine regulations Maintenance of basic services and the road network in rural areas Strengthening electricity networks through underground cabling or clearing risk areas from trees Alternative import channels for electric power Regulation of electricity for users Supporting EU and national policy, market guidance, control, taxation, and regulation Support for communication and cooperation between actors Support for improving knowledge and guidance on changes, disruptions, and risks related to impacts Increasing education Investment in information security

**Table 3:** Enablers, barriers, and benefits of key elements and critical means of food system resilience

	Enablers		Barriers		Benefits for resilience		Dimension of resilience		
							Buffer		Adaptation and (transformation)
*Key elements to enhance general resilience in food systems*									
System thinking through science and communication	Understanding of processes Research Communication Cooperation Scenario work		Plurality of drivers Complexity of feedback Narrow-mindedness Lack of experience		Ability to form a snapshot of the situation Ability to anticipate Biosafety Nutrient cycling Economic savings		×		×
Redundancy of activities and networks	Social networks Functioning markets Imports Rapidly produced compensating products		Cost effects Efficiency thinking Competition in markets		Risk diversification Secures operations through compensation		×		×
Diversity of production and partners	Policy guidance		Lack of knowledge Lack of options		Risk diversification Secures operations through diverse responses Reduces negative environmental impacts of agricultural production Increases the power to control critical inputs		×		×
Buffering strategies	Risk awareness All-year-round stores at all operating levels		Lack of knowledge Weak economic situation Difficulty in determining a reasonable level of preparedness		Secures critical operations in the event of a disruption		×		
*Critical means to enhance resilience in the Finnish food systems*									
Domestic protein crop production	Knowledge Market demand Benefits for soil productivity Suitable production conditions in Southern Finland Cooperation Policy incentives	Lack of knowledge Production risks Economic risks		Reduces dependence on imported inputs/Improves protein self-sufficiency Increases the power to control critical inputs		×		×	
Domestic renewable energy production	Regionally concentrated animal production Policy guidance Environmental statute Taxation Cooperation	Lack of system understanding Lack of political decisions Cost-effectiveness		Improved utilisation of nutrients in biomasses Reduced dependence on imported energy Additional income for farmers from manure Improved nutrient cycle in the region Can diversify crop rotation in crop farms if grass yield can be used as input Reduces nutrient strain on water systems		×		×	
Job creation measures	Identification of problem Diverse employee networks Employing young people during school holidays Technological development Long-term employment	Seasonal work High dependence on Ukrainian workers Suddenness and timing of shocks Domestic workers do not do heavy field work Labour-intensive berry and vegetable production Opposition to change practices Labour issues under many ministries		Improves the mobility of employees Secures the availability of employees Secures domestic production of vegetables and berries		×		×	

## Results

### Main disruptions and changes faced by the food system in the 2020s

The experts stressed that the disruptions faced by food systems were widespread and complex. Disruptions, combined with the identified need to transform systems in a more sustainable path, towards the circular economy, and the abandonment of fossil fuels, increased this complexity. Globally, many critical changes as well as their requirements are manifestations of humanity’s unsustainable systems and agriculture’s unsustainable production. Direct impacts of climate change such as the increase in extreme weather events, plant diseases, and pests, indirect trade disruption and political tensions, the loss of biodiversity, population growth, urbanisation, dietary changes, and vulnerable water resources cause multifaceted feedbacks in food systems that are difficult to predict.

The experts emphasised that Finland’s harsh climate meant variations in crop production between years were very large, which has led to a high production surplus in relation to consumption. The experts considered the impacts of extreme weather events on primary production to be very important (Fig. 1), reflecting the importance of the availability of domestic raw materials for industry, exports, and ultimately, consumer prices (Table 1). According to the experts, there were few infectious animal diseases classified as dangerous, and the starting point was to prevent them entering the country. Finland’s northern and remote location was an advantage in this sense, as animals moved less between different countries than in Central Europe, for example. The experts stressed that the food sector in Finland was strongly buffered by national and EU support policies. The National Emergency Supply Agency was unique in the world, monitoring critical operations through cooperation between private and public organisations.

The experts considered that the coronavirus pandemic had increased instability in international markets, with implications for the functioning of food systems. During the pandemic, Finland had managed to secure the food supply required by society. One major blow was the shortage of seasonal workers on vegetable and berry farms, a result of the sudden closure of borders in the spring of 2020. Finland lacked a sufficiently skilled workforce, and the deficit could not be fully compensated by domestic labour, which reduced the domestic production of fresh produce. At the trade level, the effects were limited to the closure of individual stores for a few hours for disinfections caused by single cases of the disease. According to the experts, the change in consumer behaviour had a greater significance in causing concerns about spikes in consumer demand that daily management would find difficult to meet. At the beginning of the coronavirus crisis, with new restrictive measures in society, fresh meat products were hoarded somewhat as a first reaction. However, the continued availability of products quickly restored consumer confidence. In addition, the rapid change in dining from institutional and restaurant catering to home food influenced food processing, distribution and trade, and sales.

The experts stressed that the food system actors in Finland were small on the European scale. In terms of volume, the food supply chain actors in industry, distribution, and trade were concentrated and strongly interdependent. In primary production, especially in the dairy and meat sectors, a large part of the volume went through a few large units, the development being strongly influenced by the efforts of companies to increase economic efficiency. This property increased food system vulnerability, because if the disruption hit critical points with a few players such as the critical imported inputs, the scope for influence might be limited because of a lack of national control, leading to notable disruptions in the food system. In Finland and in Europe, the experts highlighted, the great dependence on imported soya feed being a major risk for animal production, increasing vulnerability to trade policy disruptions. Market disruptions also threatened animal welfare, especially poultry production, because the rapid growth rate of birds was sensitive to production disruptions, and delay could result in birds not fitting processes. In addition, the illness of the workforce in a large unit or in a few large distribution channels, or the spread of an animal disease in a large processing plant, could pose a widespread threat to operations, ranging from primary production to trade, affecting food availability at the consumer end.

The experts perceived that all today’s food supply chain actors were strongly dependent on energy and electricity, and disruptions in their access could cause severe problems for food system operations. Disruptions could be severe when they affected the processing and trade of meat and dairy chains, whose products could not be placed in temporary storage. A large-scale regional power failure could especially risk food production, processing, storage, delivery, and sale. According to the experts, despite its good reserve power preparedness, primary production remained vulnerable to electricity disruptions, which endangered the supply of domestic raw materials in the supply chain. In food processing, there were fewer reserve power systems. However, the probability of simultaneous power outages in different parts of Finland was low. The retail and distribution of food were completely dependent on electricity to get products from farms or processing to storage and further to retail. In large central warehouses, robotics, a high degree of automation, and cold rooms all required electricity. Especially in densely populated cities, the impact of large-scale electricity disruptions on food security could be severe.

The functions of the food system also depend strongly on global, national, and local information and communications Technology (ICT) systems and networks. According to the experts, at worst, a cyberattack could cripple an entire digital information system and disrupt a company’s business. Food terrorism could reduce food availability and increase consumer prices, profoundly upsetting the person’s sense of security associated with food.

The experts highlighted the impacts of infectious animal diseases on food system operations as very important (Fig. 1 and Table 1). For livestock production, one of the major considered threats was the spread of African swine fever to Finland. The disruption would particularly affect pig farms, where infection would lead to the slaughter of pigs, with significant economic consequences and widespread market disruption. According to the experts, the disruption would drastically reduce the availability of domestic meat and affect meat prices in Finland. The experts considered the origin of meat was an important factor for consumers, and such a shock could thus have a major impact on consumer buying behaviour. In addition to a price increase, consumers’ distrust could be reflected in short-term changes in willingness to buy pork, and other meat and vegetable products. In the long run, the experts estimated that disruptions in livestock production would increase the popularity of the vegetarian diet.

Other notable changes and disruptions the experts mentioned were digitalisation, rapid changes in consumption, hazardous pesticide residues and genetically modified organisms, a decline in the sense of food reducing the ability to make the right food policy decisions, nuclear fallout, and war.

### Resilience means at different levels of the food system

The experts highlighted the importance of innovative people and companies with alternative initiatives, courage, and willingness to experiment with new practices as a necessity for food system resilience. Actor-specific means in primary production, processing, and retail (Table 2) which could help prevent disruptions and minimise their negative impacts were necessary to secure critical operation. The implementation of such resilience means was strongly influenced by awareness of and expertise in useful means, the financial capacity of actors to conduct actions, and communication between different actors. For example, improving soil growing conditions through diversification required an understanding of complex biological feedbacks. Information security was an example of an expensive resilience means that required special knowledge and for which larger companies usually had better abilities to prepare for. Control of infectious animal diseases required the communication and cooperation of different actors.

The experts noted that for new and unexpected disruptions, the lack of information on the necessary means increased costs. During the Covid crisis, the food processing sector prepared for a collapse in the number of employees because of increased illness, and massive measures were taken rapidly to prevent the spread of the disease. Due to effective prevention measures throughout society, the effects of Covid did not threaten food availability.

Society plays an important role in enabling an encouraging operating environment for innovations and securing critical inputs and products during market disruption. The food system experts considered monitoring of national and international disruptions and food security indicators important. In Finland, the National Emergency Supply Agency monitors the state of critical sectors, including primary and food production, acting as an important buffering system for the national food supply. The experts highlighted its role in facilitating communication between the private sector and the authorities, and in supervising and giving guidance during the crisis. At policy level, both national and EU agricultural policy aims to maintain food production throughout the EU, but policy has only a few means to enhance resilience.

The experts highlighted the importance of social networks enabling the communication and cooperation of food system actors. In exceptional circumstances, network communication enabled the formation of a snapshot in the event of a disruption, allowing information sharing, quick decision making, and processes to be changed. In normal circumstances, communication between the private and public sectors was important for building mutual trust between actors and enabling companies to obtain information about risks nationally and internationally, share the knowledge different actors had, and create innovations. In the food system, vulnerability to various disruptions required the continuous development of preparedness for new disruptions from primary production to industry and trade.

### Means to enhance the resilience in Finnish food system

We identified three means of enhancing resilience in Finnish food systems: (1) domestic production of protein crops; (2) domestic renewable energy production and strengthening of electricity transmission network; and (3) job creation measures. These means would implement the first three resilience elements presented above, strengthening the future stability of food supply.

#### Domestic production of protein crops

The diversification of cultivation through the production of protein crops would directly reduce the food system’s vulnerability to disruptions from imported inputs. At farm level, diversification would improve soil growing conditions and improve the stability of farming, which was important in the face of climate change.

According to the experts, climate change, especially the extension of the growing season, increasingly enabled the diversification of crop production with protein crops. For example, the production area under oilseed rape had quickly grown during the twenty-first century, and climatic conditions were also beginning to be favourable for fava bean cultivation. The diversification of cultivation within the same year was key, requiring the farmer to produce sufficient volumes of different crops. Larger farms increased the farmer’s opportunities to find suitable agricultural plots for crops that enhanced diversity. The experts considered that a sufficient arable area in Finland would enable the self-sufficient production of protein crops. Succeeding in diversified farming required new skills of farmers. The farmer faced a higher production risk with special crops than with traditional cereals due to the lack of experience, and the higher price did not necessarily take this risk into account yet. Advisory, farmer peer support, and decision-making tools could increase knowledge and encourage diversification. The experts stressed that there was a demand and will in the food industry to increase the use of domestic raw materials, despite the adaptation requirements for batch size and crop species in the production processes which were currently planned for imported soya.

#### Domestic renewable energy production and strenghtening of electricity network

Increasing the production capacity of domestic renewable energy was considered to improve energy self-sufficiency. This was important because dependence on electricity was continuing to increase in society. Particularly during consumption peaks in the cold winter months, domestic production would reduce the risk of having to regulate electricity. Strengthening electricity transmission connections with abroad to enable both to import export electricity was considered important. Strengthening electricity networks through underground cabling and clearing risk areas to prevent trees from falling on power lines were ways to secure energy access.

Increasing the energy self-sufficiency of farms by increasing biogas production was considered to create environmental and economic benefits. Farms’ own energy production through bioreactors could even enable energy to be sold offsite. On livestock farms, manure could be processed into energy and as a more concentrated nutrient, which would improve nutrient recycling, and could reduce water and climate emissions. Grass yields could also be utilised as feed in the bioreactor, which could promote the use of grass as part of crop rotations on crop farms. This would reduce dependence on imported energy. The construction of biogas plants required an economically viable market-based operating environment. Agricultural, energy, and climate policies played an important role in creating incentives and a predictable market environment, for example, through taxation and environmental legislation.

#### Job creation measures

In Finland, the seasonality and heaviness of work and the low level of pay reduced domestic labour interest in farm work. Finnish farms also preferred to employ motivated and skilled employees from abroad. For foreigners, the salary level in Finland was high in relation to their home country. The experts suspected that new arrangements and the replacement of foreign with domestic labour would inevitably increase costs. Employing young people during school holidays was suggested as a potential solution. Long-term employment year after year was considered beneficial for both the employer and employees. The availability of domestic labour was affected by the number of young people and the unemployed and on the other hand, by the concentration of labour-intensive production. Adequate access to domestic labour was particularly difficult for labour-intensive berry and open-air vegetable farms far from population centres.

Labour networks that would extend to several countries would improve opportunities to get foreign employees to work in farms. The experts also highlighted the need to facilitate the mobility of foreign employees to be able to work without interruption in different workplaces. This could safeguard farms’ labour needs and prolong the employment relationships of foreign workers in Finland. Technological development could somewhat reduce vulnerability by facilitating and lengthening the harvesting periods on berry farms, for example. The authorities played a key role in establishing rules and procedures for quarantine practices and mobility to simultaneously ensure the adequacy of the workforce and prevent the spread of disease. At the administrative level, close cooperation between different ministries could improve such work.

### Key elements of resilience in food systems

**Fig. 2:**
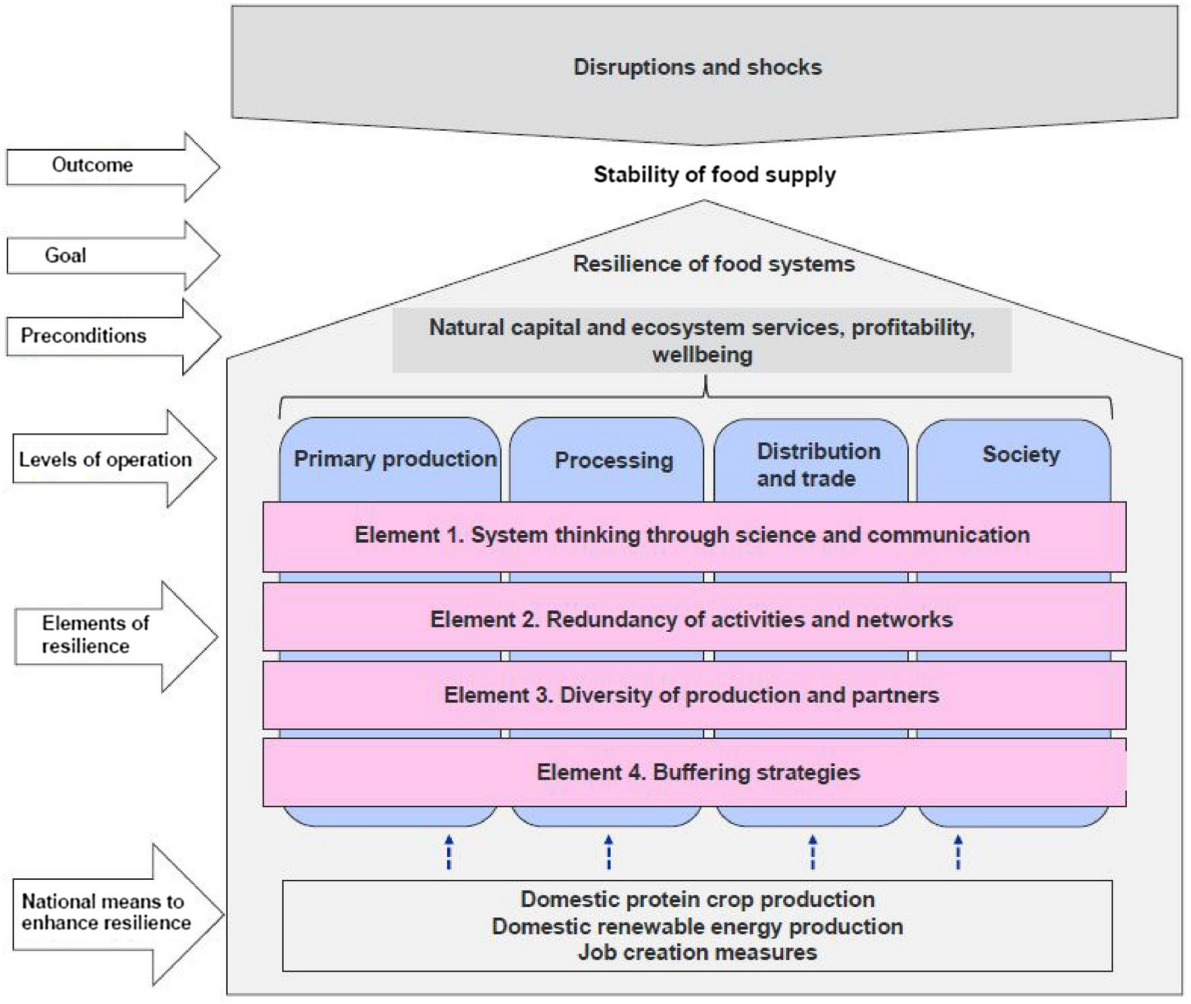
Key elements of resilience securing stability of food supply in the event of disruptions and shocks

At the food system level, we identified four key elements of resilience based on the expert interview data: (1) system thinking through science and communication; (2) the redundancy of activities and networks; (3) the diversity of production and partners; and (4) buffering strategies (Fig. 2).

### System thinking through science and communication

The identification of critical dependencies and an increasing understanding of complex interactions and feedbacks were emphasised as important for resilience. For example, for infectious diseases, risk preparedness started with identifying risks and adhering to good practices, including hygiene, protective clothing, and preventing people entering production facilities. The experts emphasised the importance of acknowledging that a perfect anticipation plan could not be pre-emptively tailored to mitigate the impacts of a sudden disruption. Planning must therefore be approached as a reactive tool, allowing the identification and rapid implementation of targeted measures for a specific situation. The experts considered multidisciplinary system analysis important for the identification of critical feedback in different sectors. National and international networks and cooperation were important means of advancing provision for various disruptions. Active communication between actors at horizontal and vertical levels of the food system, such as the private sector and public authorities, enabled the formation of situational awareness in the event of a disruption and an understanding of the boundary conditions of the activities of various actors. All this could enable the identification of critical risks and rapid reaction, which could help stop the spread of the disruption and help solve the problem (Table 3). In Finland, experiences of cooperation between business competitors had been positive during disruptions. Redirecting raw materials and the reorganisation of processing were examples of working together for a common goal to prevent large-scale problems. As the operating environment was constantly changing, new innovations and co-development promoting adaptation were needed. Cooperation between actors was important for the success of actor-specific initiatives and for gaining environmental, social, and economic benefits.

For new disruptions such as the problem in seasonal labour supply resulting from border closures during the Covid-19 pandemic, cyber incidents and African swine fever (ASF), the lack of previous experience hindered the understanding of the existing situation and consequences, and the selection of the right measures. At worst, this could lead to an expansion of disorder.

### Redundancy of activities and networks

The experts considered that a wide network of actors, market partners, and activities distributed risks directed at food system operations. For example, extensive import channels would reduce dependence on a single supplier, securing the availability of raw materials and critical inputs in the event of disruptions, when the other actors could compensate for the functions one that was disrupted. In Finland, this was extremely important for the processing industry, because more than half oil plant raw materials were imported, for example. Imports and exports were important, because they diversified the selection of goods and ensured the availability of raw materials, both in the home market and abroad in the event of disruptions.

Geographically extensive primary production ensured the availability of domestic raw materials in extreme weather events in certain locations. In the event of disruption to specific production, such as a collapse of domestic pig meat production caused by ASF, alternative meat products would help compensate losses. These could be either imported pig meat or other domestic meat products such as poultry. Plant proteins could also compensate for the missing meat products. For labour, extensive networks would secure the availability of employees, and thus the maintenance of primary production on farms. It would also secure domestic raw material flows from farms to industry. An extensive retail network would secure consumers’ access to food during cyberattack directed at the retail trade, for example.

### Diversity of production and partners

The experts stressed that at farm level, the cultivation of different crops and varieties within the same year on different plots would secure yields despite disruptions (Table 2) and thus increase the resilience of cultivation systems. Having plants at different stages of development would ensure the success of cultivation. For example, severe drought and high temperatures could hit a critical development point for spring cereals and cause significant crop losses. At the same time, however, oilseeds were just evolving and were less vulnerable to such disruption. Similarly, the pea was not sensitive to drought; on the contrary, it became lusher. However, autumn cereals usually had had time to develop deep roots when dry and warm periods came to Finland, so their growth was not disturbed.

The experts also emphasised that high crop diversity in crop rotation would promote good soil health, which buffered negative impacts of climate change such as heavy rainfall and drought. Increasing diversity, especially by including grasses, oilseeds, and protein crops in crop rotations in southern Finland, could improve the adaptability of agriculture to climate change. In Finland, the high production surplus of cereals in relation to consumption would enable the increase of production of protein crops in the existing cereal area. This would reduce the dependence on imported protein and add value to the domestic plant and animal protein production because of the reduced use of soya.

It was emphasised that diversity between farms within a region was important for securing domestic production for industry. In addition, the diversity of agricultural sectors was highlighted. In Finland, a strong livestock sector, based on grassland production in northern Finland, had secured national food security in past decades and centuries, compensating for possible losses in crop production in the south during crop failure years. Especially when the growing season was very rainy and cool, the production of special crops and cereals was risky in Finland. More diverse agricultural production at regional level would globally reduce the negative environmental impacts of highly concentrated industrialised agricultural production, such as the production of soya in South America. The experts considered economic incentives most efficient for increasing the diversity of food production.

### Buffering strategies

The experts stressed that buffering strategies and backup systems were important to secure raw material flows and the availability of critical products at different levels of the food system, from primary production to consumers. The public authorities play the leading role in enhancing these strategies. They also call for committed cooperation between food system actors.

Biosecurity, HACCP (hazard analysis and critical control points) and contingency plans were considered important for protecting critical operations from damaging organisms and as guidelines for action in the event of the risk materialising. At all food system levels, hygiene was considered important for the prevention of infectious diseases. Regarding infectious animal diseases, preventing the spread of diseases was a priority. In Finland, the long shared land border with Russia to the east made it more difficult to control African swine fever, which spread with wild boar (*Sus scrofa*), while in the south and west, the sea prevented the movement of animals.

A high production buffer, that is, a production surplus in relation to consumption, for cereals balanced crop variability between years, which was important with increasing extreme weather events. Compared to many other European countries, Finland had a remarkably large year-round stock. Maintaining physical reserve stocks ensured the availability of critical inputs and raw materials for food supply. Especially at the farm level, storage capacity was large for cereals to obtain the best market price. Industry and trade also had large stocks. In addition, society’s reserve stocks increased the buffer against disruption. In Finland, the National Emergency Supply Agency facilitated communication between the private sector and the authorities, supporting the maintenance of security of supply in society. In a crisis, the work also involved supervision and guidance.

Backup connections for distribution secured the availability of electricity. Reserve power for different parts of the food chain was important in the event of power outages, especially in rural areas, where there were fewer electricity grids. However, backup power was very expensive, and the probability of a widespread power outage in Finland was considered low, which reduced actors’ interest in acquiring reserve power. Backup systems for ICT connections and payment systems were also very important as the digitalisation of the food system progressed.

## Discussion

Complex food system disruptions cause multifaceted feedback and consequences which are difficult to anticipate. We identified the vulnerabilities of Finnish food system to disruption, and the means to enhance food system resilience by conducting semi-structured in-depth interviews with food system experts.

The experts highlighted that the interconnected disruptions and changes threatening the stability of food supply mostly affected primary production. This stemmed from Finland’s food supply strategy, which was strongly based on domestic primary production, in contrast with neighbouring Sweden, for example (Bovin 2018). The direct impacts of climate change, increasing extreme weather events, and indirect effects causing market disruptions fell on farms. Due to poor economic profitability, farms had the lowest capacity in the food system to respond to disruption (Himanen et al. 2016). The low profitability of primary production resulted in insufficient investment in the growing condition of fields, machinery, and infrastructure. In addition, ageing farmers (Torvelainen et al. 2020) and their poor wellbeing threatened economic, ecological, and social sustainability and resilience. The importance of infectious animal diseases was emphasised in our study, probably because the coronavirus pandemic was at its height when the research data were collected.

In the current food system, we can identify many operators that enhance the resilience, such as cooperation through the National Emergency Supply Agency that coordinates the management of critical sectors and ensures the sufficient storages of production inputs, and the high level of self-sufficiency in primary production. However, there are also areas to be developed. The homogenous agriculture and the regional distribution of production, as well as the concentration of both processing and trade, increase the vulnerability to sudden disturbances. It is difficult to implement large structural changes to the functions of the food system in an instant. The current situation has been developed over decades as a result of policy guidance and pursuit of efficiency. Moreover, actors in the food system possess different capabilities to partake in and adapt to structural changes causing temporal and spatial variation to developments in different parts of the food system. Based on the analysis of the data we highlight the following elements as important for promoting resilience:

### Resilience element 1: system thinking through science and communication

Although infectious animal diseases have previously been observed as a major threat to the functioning of food systems (Graham et al. 2008; Roe et al. 2020), the consequences of the Covid-19 crisis related to problems with the availability of foreign labour on vegetable and berry farms, and the hoarding behaviour of consumers were unforeseen. This is an important finding in understanding the complexity of the impacts of disruptions in food systems, highlighting the importance of system thinking. A systemic perspective in science and research can help identify critical components and interactions (Kasper et al. 2017). A food system approach (Piters et al. 2021) could provide useful tools for addressing critical socioeconomic and environmental aspects that influence food security and cause vulnerabilities, and in searching for elements and means to strengthen food system resilience.

Pettit et al. (2010) described collaboration in the supply chain context as referring to the ability to work effectively with other actors for mutual benefit in forecasting and risk management. In line with this, the experts in our study highlighted the importance of communication, cooperation, and trust between actors in sharing knowledge and clarifying the situation, both for risk prevention and adaptation. A novel finding in our study was that high concentration and interdependence in the food system increased dialogue. This improved knowledge sharing and system understanding, strengthening the ability to respond to disruptions. This result broadens knowledge of the relationship between efficiency and resilience, because in the resilience context, one is accustomed to thinking that specialisation and the regional concentration of primary production only weakens the redundancy of elements and thus creates vulnerabilities. Multi-stakeholder platforms (MSPs) could stimulate partnership between different actors through knowledge exchange, joint learning and co-creation and speed up the implementation of research results (van Ewijk and Ros-Tonen 2021; Ros-Tonen et al. 2015; Klerkx et al. 2012).

### Resilience element 2: redundancy of activities and networks

The existence of similar organisms, actors, and activities that can fully or partly replace each other provides insurance for the food system by enabling the compensation of elements (Rosenfeld 2002; Chapin et al. 2009; Biggs et al. 2012, 2020). The development in food systems in recent decades does not support the strengthening of functional redundancy. The reduction in the number of farms and development towards concentration at all levels of food systems as a result of the intensification of production, boosted by agricultural policy (Czyżewski et al. 2021), reduces options. Imported soya feed is a good example, because its production is concentrated in only a few areas outside Europe (FAOSTAT 2021), increasing the risk of major food system disruption. The maintenance of an extensive network of market partners should therefore also be encouraged to secure critical imported inputs in the event of disruption. This promotes flexibility, facilitating response to change (Tukamuhabwa et al. 2015). Expanding the production areas of protein crops in Finland would reduce dependence on imports. The existing arable area would enable self-sufficient protein crop production. Such a development would require a commitment from all the actors in the food systems, including cooperation between actors, economically viable markets, and an encouraging political climate. Domestic renewable energy production contributes both food system, and larger societal resilience. Agricultural biogas production is often highlighted as a key mean in unlocking synergies between the goals in energy and agricultural policy domains, enhancing redundancy in energy production and diversifying income and product portfolios of agricultural enterprises (Winquist et al. 2021). From the energy system’s perspective, biogas production enables flexible generation of electricity which allows both production and demand based balancing options for intermittent wind and solar power, as well as inflexible nuclear production in the Finnish energy system. Agricultural enterprises, in turn, would strengthen their economy through reduced impact from energy price outages and price hikes, generate added revenues from energy sales, generate on-farm buffers for energy related disturbances.

### Resilience element 3: diversity of production and partners

The diversity of species, varieties, and production sectors was considered important for food system resilience. This is in line with previous studies (e.g. Darnhofer et al. 2010a, b; Cabell and Oelofse 2012; Carpenter et al. 2012; Hodbod and Eakin 2015; Hertel et al. 2021). In primary production, increasing diversity secures the harvest and promotes both short- and long-term resilience (Degani et al. 2019). Different reactions to change are important for resilience—for example, in different ways to respond to drought (Elmqvist et al. 2003; Folke et al. 2004). Differences in yield responses increase resilience to different and unexpected weather conditions (Hakala et al. 2012; Kahiluoto et al. 2014, 2019) and provide adaptation options for climate change (Howden et al. 2007). It is important to include different crops and varieties in the crop rotation in the same year, either as pure crops or as mixtures. The diversification of crop rotations, and in particular the inclusion of protein crops in the rotation, would improve soil growing conditions and enhance stability in cultivation, increasing the buffering and adaptive capacity of agriculture for different weather phenomena. Differences in the behavioural responses of actors in the food supply chain have also been identified to improve resilience in market disruption situations (Kahiluoto et al. 2020). The spread of agroecological practices plays a crucial role in how well farmers adapt to climate change (Altieri et al. 2015). To diversify the agricultural production the communication with food processors is important as they define the quality standards for the raw materials they buy.

### Resilience element 4: buffering strategies

The food supply in Finland is strongly based on domestic agricultural production. The degree of food self-sufficiency is high compared to neighbouring industrialised countries (Eriksson and Peltomaa 2017). In Finland, the security of supply of critical production inputs is ensured in cooperation with actors in the public, private, and civil sectors. The participation of actors in the private sector is partly voluntary and partly statutory (National Emergency Supply Agency 2020).

The harsh climate, remote location at the outer edge of Europe, and memories of the time of scarcity during World War II explain the food supply strategy in Finland. Good cooperation between different actors is probably the result of post-war reconstruction. With increasing uncertainty in the world, it is becoming increasingly difficult for markets to maintain basic functions (Sharma et al. 2020). To strengthen the resilience, it is important to strengthen the reserve stocks of critical inputs and also to share experiences of the good practices with other countries.

## Conclusions

The interconnected disruptions and changes threaten the stability of food supply, and they are mostly affecting primary production. System thinking through science and communication, redundancy of activities and networks, diversity of production and partners and buffering strategies are key elements to enhance the general resilience in the food system.

1. Versatile, self-sufficient, and profitable primary production plays a key role in food system resilience

Primary production is the cornerstone of the entire food system, and the shocks and disruptions it faces therefore call for a sufficient, versatile, and stable domestic production volume, supported by the available domestic renewable energy. To enhance resilience, increased self-sufficiency in protein crops would decrease the food system’s vulnerability to market fluctuations of imported feeds. Increasing protein crops could also improve crop rotation, which is poor in many places, especially on crop farms due to the reduction of livestock, resulting in monocultures in cultivation and a decrease of organic matter in the soil. Improving diversity and soil fertility is one of the most important ways to prepare and adapt for climate change at farm level. Diverse farming could also increase the level of carbon storage in the soil, increase biodiversity, and improve yields.

2. Cooperation based on trust builds shared understanding, a willingness to take responsibility, and responsiveness during sudden shocks and disruptions

Dialogue and cooperation based on trust between different actors in the food system provide an understanding of disruptions and their impacts, enabling the creation of innovations to combat disruptions and adapt to changes in the operating environment. To a certain extent, concentration and interdependence in the food system increase dialogue and cooperation. In the event of a disruption, cooperation enables the formation of a situational picture, enabling the rapid response and efficient communication that is important for maintaining critical core functions. Sufficiently large and diverse social networks reduce the risk of the effects of market failures. The strengths of the Finnish food system are the operating plans of security of supply organisation, safety stocks, and good cooperation and trust between operators. This is a good starting point for finding solutions for the unexpected vulnerabilities which are identified after new shocks, such as ensuring the mobility of foreign labour. There is however need for a broader partnership in the food system, including besides food supply chain actors also consumers to promote knowledge and co-creation. For this multi-stakeholder platforms could provide ideas which should be investigate further.

3. Stable food availability calls for organised security of supply

In terms of critical resources, sufficient reserve stocks buffer disruptions over a short period in the event of unexpected production or market disruptions. For critical inputs and cereals, the existing buffering strategies increase flexibility in the Finnish food system. Increasing the production capacity of domestic renewable energy would secure access to energy, which would further contribute to stable food availability. It could also improve nutrient recycling and reduce nutrient losses.

4. Resilience perspective should be integrated into food system strategies and guiding policies

Increasing disturbances directed to food systems highlights the importance for comprehensive food policy. The systemic resilience elements and concrete measures should be more strongly involved in the strategies and future policies that guide the food sector. Introducing and strengthening the identified resilience elements and means in the food system call for the preparation of a more holistic and coherent food system policy that acknowledges and emphasises resilience alongside efficiency. Together, resilience means contribute both to resisting disruptions and taking steps towards a more sustainable future by getting rid of the fossil economy.

It should be noted that the results in this article are based on rather small number of interviews, and in further studies the development of resilience could be based on integrating qualitative and quantitative methodologies i.e. using mixed research studies as the foundation for the future research (Leech et al. 2010).

## Supplementary Information

Below is the link to the electronic supplementary material.

Supplementary file1 (DOCX 46 kb)

## Data Availability

The data that support the findings of this study are available from the corresponding author, [Karoliina Rimhanen], upon reasonable request.
