# Supplementary material for: The elements of resilience in the food system and means to enhance the stability of the food supply
Source: Environ Syst Decis. 2023 Jan 2;43(2):143–60. doi: 10.1007/s10669-022-09889-5 (PMC9806810; doi:10.1007/s10669-022-09889-5)
Supplement: Supplementary file 1 — Supplementary file1 (DOCX 46 kb) [file 10669_2022_9889_MOESM1_ESM.docx]

Supplementary material 1

**The elements of resilience in the food system and means to enhance the stability of the food supply in Finland**

**Interview guide**

1. Could you briefly introduce yourself and your expertise in the food system?
2. What do you think are the main changes, disruptions, and shocks to the food system now, in the 2020s?
3. How significant can the following disruptions be in ensuring food security? Rate on a scale of 1-5.

| Example of disruption | 1=not important at all | 2=of little importance | 3=of average importance | 4=fairly important | 5=very important | 6= I can not say |
| --- | --- | --- | --- | --- | --- | --- |
| A policy disruption such as a trade policy conflict that directly affects the market |  |  |  |  |  |  |
| Gradual climate change and climate shocks such as an increase in extreme weather events |  |  |  |  |  |  |
| Market and price disruptions such as fluctuating or high input and product prices, investor uncertainty |  |  |  |  |  |  |
| Changes in the availability of resources such as available arable land, labour, or feed |  |  |  |  |  |  |
| Disruptions in energy supply, for example, in power distribution |  |  |  |  |  |  |
| Cybersecurity threats such as disruptions to electronic and networked systems and information security |  |  |  |  |  |  |
| Bioterrorism related to biological weapons such as pathogenic microbes and toxins |  |  |  |  |  |  |
| Infectious animal diseases such as African swine fever or Covid-19 |  |  |  |  |  |  |
| Contaminated foods that cause food poisoning such as salmonella |  |  |  |  |  |  |
| Unexpected “black swans”, i.e. highly unlikely events |  |  |  |  |  |  |

1. Would you like to add something?

Next, we select from these examples 1-3 for closer examination.

- Political disruption: There is a social conflict, as a result of which the import of very important inputs is prevented to Finland.
- Climate-related shock: Extreme droughts is faced in the growing season and crop yields collapse.
- Market disruption: The price of an important input rises surprisingly fast.
- Changes in the availability of resources: Due to the Covid-19 epidemic, foreign workers are prevented from entering Finnish vegetable and berry farms.
- Disruptions in energy supply: The storm will interrupt the supply of electricity for several days.
- Cybersecurity threat: A cyber-attack paralyzes key system functions and jeopardizes animal welfare.
- Bioterrorism: The food system is the target of a terrorist attack in which the food chain is intentionally contaminated with a biological agent or poison.
- Infectious animal diseases: Dangerous and easily spreadable animal disease spreads to humans from primary production
- Contaminated foods: Food contaminated with bacteria (e.g., Campylobacter, Salmonella, Shigella, or EHEC) causes a widespread food poisoning epidemic.
- Unexpected “black swans”

1. How does the selected disorder affect the functions of the food system?
2. How to prepare for a disturbance? Are we preparing for this now?
3. Is preparation expensive or cheap? How and to whom are the costs of preparedness incurred and how large are they?
4. What promotes preparedness? What measures help to prepare for disruptions in the food system and where in the food system should they be targeted?
5. What issues make preparedness difficult?
